# How to forget a “traumatic” experience: a case report of transient global amnesia after nasopharyngeal swab for Coronavirus disease 19

**DOI:** 10.1186/s12883-021-02295-5

**Published:** 2021-07-05

**Authors:** Sabrina Ravaglia, Antonio Zito, Lara Ahmad, Isabella Canavero

**Affiliations:** 1 IRCCS, Mondino Foundation, 27100, Pavia, Italy; 2 Department of Brain and Behavioral Sciences, University of Pavia, Via Agostino Bassi 21, 27100 Pavia, Italy; 3 Cerebrovascular Diseases Unit, Fondazione I.R.C.C.S. Istituto Neurologico Carlo Besta, Milan, Italy

**Keywords:** COVID-19, SARS-COV-2, TGA, Nasopharyngeal swab, Case report

## Abstract

**Background:**

Transient global amnesia (TGA) is a clinical syndrome characterized by a temporary short-term memory loss with inability to retain new memories, usually lasting 2 to 8 h. TGA may be related to several medical procedures, including angiography, general anesthesia, gastroscopy.

**Case presentation:**

We report a 58-year-old woman who experiencing TGA one hour after the execution of her first-time nasopharyngeal swab for COVID-19. Brain MRI showed a typical punctate Diffusion Weight Image (DWI) hippocampal lesion.

**Conclusions:**

This is the first report of TGA after the execution of nasopharyngeal swab for COVID-19. This association lengthen the list of medical procedures associated with TGA, and we discuss the possible plausible mechanisms by which a nasopharyngeal swab could trigger TGA.

## Background

Several medical procedures have been found to be associated with transient global amnesia (TGA), With cerebral and coronary angiography as the most common antecedents [1].

Proposed triggers are the same as non-procedure-related TGA and include acute pain, migraine, Valsalva maneuver-associated activities, and different stress-related events. These latter, including emotional, psychological, and physical stress, are thought to influence hippocampal status through several different mechanisms, ultimately leading to neuro-inflammation, oxidative stress, and neurotoxicity [2]. The characteristic memory disturbances and hippocampal lesions are thought to result from non-specific vasomotor changes (vasospasm, hippocampal hypoperfusion) or from peri-procedural use of specific drugs, peculiarly affecting susceptible, memory-relevant structures of the temporal-limbic circuit [3].

The nasopharyngeal swab is usually perceived as uncomfortable and produces a bad memory in the tested population; it is the gold standard technique for diagnosing Coronavirus disease 19 (COVID-19) active infection, and several millions of tests are being performed. Adverse effects include short lasting pain, epistaxis, headache, ear and nose discomfort, rhinorrhea [4].

Disproportionate terrors about the swab are somehow fueled by media and sensationalistic journalism: people who have had the COVID-19 swab test say it feels like their “brain is being pierced by an oversized cotton bud”. A case of cerebrospinal fluid leak has been recently reported, pertaining a patient with a previously undiagnosed skull base defect at the fovea ethmoidalis [5].

Nasopharyngeal swab has not been previously reported among the procedures associated with TGA, likely because nasopharyngeal swab was not such a common procedure until these latter months.

## Case presentation

We report of a 58-year-old woman, who did not suffer from any pre-existing medical conditions, with the only exception of being mildly overweight (BMI 31.25). In particular, she did not smoke, she had no history of migraine, and she did not receive any hormone replacement therapy after menopause, that occurred at age 51. One hour after the execution of her first-time (later revealing as negative) nasopharyngeal swab, while driving home, she likely experienced amnesia: the bystanders reported that the woman pulled over the car, lowered the window and called for rescue, telling she was unable to find the right direction; when she was asked where she was going, she answered she did not remember and began to behave anxiously. The bystanders reported that she could tell her name and other personal information, and that she could herself remember the unlock code of her phone and call her husband. The husband noticed she was disoriented, repeatedly asking the same questions about where she was and what she was doing. This episode lasted for about two hours then reverted; the day after, she was conducted to our attention for evaluation. A memory gap for the episode persisted: she could remember having had the swab tested, then having paid for the test, having left the hospital, and reached the car after the procedure, but she could not remember the following events, including those witnessed by the by-standers and including the call to her husband and its arrival on the place where she had left her car.

**Fig. 1:**
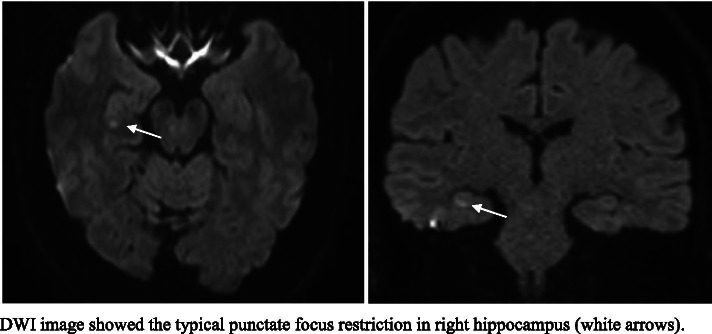
Brain MRI

Neurological examination was normal. Brain MRI, performed one day after TGA, revealed a hippocampal punctate area of DWI restriction (Fig. 1), confirming the clinical suspect. Supra-aortic vessels Duplex ultrasonography and EEG were negative, ruling out mimics. The strict temporal relationship between nasopharyngeal swab and TGA may have been simply coincidental, but we are usually prone to investigate each time carefully the triggers for TGA, since TGA recent anamnesis is often full of peculiar, even bizarre, but usually biologically fitting antecedents.

## Discussion and conclusions

We believe that the swab may have acted as a TGA trigger, via a series of potentially plausible mechanisms:

1. First, direct touch stimulation of the lower nasal and pharyngeal structures, innervated by the sensory terminations of the Xth nerve, may cause cerebral vasomotor phenomena via central vagal nuclei, which are known to be involved in sympathetic cardiovascular regulation.
2. Second, the procedure itself may be intended as a Valsalva-like maneuver: indeed, the nasopharyngeal swab could cause a temporary stop at the glottis, or, even more peripherally, at the nasopharynx or at the nostrils.
3. Last, the swab is often loaded with emotional stress, both directly related to the local pain and discomfort, and, in the pandemic context, also more generically related to worrying for the result and its practical consequences. Indeed, stress-related triggers (including emotional and psychological stress) my lead to hippocampal dysfunction via several mechanism, involving glutamate neurotoxicity, release of vasoactive stress hormones such as angiotensin I and II, and oxidative stress [2].

With this note, we would suggest that nasopharyngeal swab might lengthen the list of medical procedures associated with TGA, by multiple potential mechanisms. In our patient, the free time-interval between the procedure and the onset of TGA supports the occurrence of an indirect mechanism, such as emotional stress and pain, rather than Valsalva-like or direct vasomotor effects via the vagus nerve. Indeed, direct pain to the brain parenchyma is believed to have a role even in TGAs following cerebral angiography, that is by far the most common procedure associated with TGA.

Reluctantly paraphrasing sensationalistic magazines, we suggest that a nasopharyngeal swab can, in fact, “pierce your brain”, or rather your hippocampus, in the form of a short-time memory loss: perhaps that is what you need to forget the swab and this pandemic emergency, Freud would have said.

## Data Availability

The data that support the findings of this study are available on request from the corresponding author. The data are not publicly available due to privacy or ethical restrictions.

## Abbreviations

TGA: Transient global amnesia
COVID-19: Coronavirus disease 19
DWI: Diffusion Weight Image
